# Dietary diversity practice and associated factors among adolescent girls in Dembia district, northwest Ethiopia, 2017

**DOI:** 10.1186/s40985-020-00137-2

**Published:** 2020-10-09

**Authors:** Kedir Abdela Gonete, Amare Tariku, Sintayehu Daba Wami, Temesgen Yihunie Akalu

**Affiliations:** 1 Department of Human Nutrition, Institute of Public Health, University of Gondar, Gondar, Ethiopia; 2 Department of Environmental and Occupational Health, Institute of Public Health, University of Gondar, Gondar, Ethiopia; 3 Department of Epidemiology and Biostatistics, Institute of Public Health, University of Gondar, Gondar, Ethiopia

**Keywords:** Adolescent, Dietary diversity, Dembia district, Ethiopia, “low- and middle-income countries”

## Abstract

**Background:**

Dietary diversity is defined as the number of food groups or items consumed over a reference period, and usually, it is a problem in developing countries including Ethiopia. Inadequate dietary diversity is one of a major public health problem and can result in physical, emotional, and psychological changes among adolescents. However, studies on dietary diversity among school children were very limited. Hence, this study aimed at determining dietary diversity practices and factors among adolescents in Dembia district.

**Methods:**

A school-based cross-sectional study was conducted from March 1 to April 15, 2017, at Dembia district, northwest Ethiopia. A total of 474 study subjects were selected using the multi-stage sampling technique. A structured and pre-tested questionnaire was used to collect the data. Dietary diversity was measured through standard tool adopted from Food and Nutrition Technical Assistance (FANTA) 2016 using the 24-h recall method. A multivariable binary logistic regression model was employed to identify factors associated with a diversified diet.

**Result:**

This study illustrated that 32.3% (95% CI 27.9–36.8) of the adolescents had adequate dietary diversity. Inadequate dietary diversity was significantly associated with being Muslim (AOR = 0.3; 95% CI 0.1–0.7), self-employment (AOR = 0.3; 95% CI 0.1–0.9), middle (AOR = 0.5; 95% CI 0.3–0.8) and high wealth category (AOR = 0.3; 95% CI 0.2–0.6), and underweight (AOR = 3.5; 95% CI 1.3–9.5).

**Conclusion:**

The findings of this study showed that only one-third of adolescent girls have adequate dietary diversity. Low level of dietary diversification suggested points to the need for strengthening efforts targeting to improve the healthy dietary practice of adolescents by giving due attention to poor households and undernourished adolescents.

## Background

Adolescents constitute a significant portion (1.2 billion) of the world population, and they are marginalized and disempowered groups in many contexts. They commonly lack a voice on the social stage, have constrained access to resources, are likely to drop out of school, and remain vulnerable to exploitation and violence [1]. Adolescence is a critical period determining current and future behaviors. Hence, behavioral interventions, including on dietary issues, at this stage will likely help bring intended change during adolescence and throughout their lifetime [2–4]. Adolescence also offers a window of opportunity to achieve optimal growth and development and delay or prevent the risk of non-communicable diseases [5].

Therefore, ensuring healthy dietary habits has paramount importance during adolescence as a poor diet also predisposes to poor physical and cognitive development [6, 7]. Dietary diversification is one way of enhancing healthy eating behavior and nutrient adequacy of the diet [8–10]. Nevertheless, the majority of adolescents are undernourished and have an undiversified diet in low- and middle-income countries [11]. As an illustration, a monotonous cereal-based diet of adolescents is reported by former studies [12, 13]. The majority of world adolescents (90%) live in low- and middle-income countries. In some countries, nearly half of all adolescents are stunted [11].

Dietary diversity is defined as the number of food groups or items consumed over a reference period. It can be measured at a household or individual level through the use of a questionnaire. Most often, it is measured by counting the number of food groups rather than the food items consumed. Although the reference period can vary, it is most often the previous day or week [8]. Dietary diversity is significantly associated with micronutrient adequacy [9]. High dietary diversity is generally considered to be a positive feature in a diet [10], and in some low- and middle-income countries, the diversification of diet is promoted through food-based dietary guidelines [14].

Lack of dietary diversity is a serious problem among the poor populations of developing countries, where diets are based predominantly on starchy staples which lack essential micronutrients and contribute to the burden of malnutrition and micronutrient deficiencies [12, 13]. More specifically, women in the reproductive age group, adolescents, and children are most vulnerable to malnutrition due to low dietary intakes, inequitable distribution of food within the household, improper food storage and preparation, dietary taboos, infectious diseases, and health care [15].

Moreover, poor health and inadequate diet adversely affect not only the health and physical growth of school children, but also their intellectual capacity, social skills, and academic performance [6, 7].

In Ethiopia, the Ministry of Health became aware of the poor dietary habit of adolescent girls, consequently and considered dietary diversification as one strategy to ensure the nutrient intake of children. Although the Ethiopian Government has tried to intervene through micronutrient supplementation and health education for infants, young children, pregnant, and lactating mothers, there has not been an intervention program for adolescent age groups for strengthening nutritional status [6].

As a result, regularly investigating the dietary intake of adolescents is vital to monitor the efforts in place. However, literature is scarce, including the current study area. To this effect, this study aimed to investigate dietary diversity and its associated factors among high school adolescent girls in Dembia district, Ethiopia.

## Methods

### Study design and settings

A school-based cross-sectional study was conducted from March 1 to April 15, 2017, among late adolescent girls aged 15 to 19 years in Dembia district. Dembia is located at the Amhara Region which is 765 km from Addis Ababa, the capital of Ethiopia. The district has 7 high schools and 135 elementary schools in 45 kebeles (smallest administrative units), and a total of 5071 adolescent girls were attending high schools in Dembia district.

### Study populations, sample size determination, and sampling procedure

All adolescent girls attending high school in Dembia district were the target population. The sample size was calculated using the single population formula by taking the proportion of dietary diversity practice as 26.8% [16] and assuming a 95% level of confidence, and a 5% margin of error. Furthermore, a 5% non-response rate and a design effect of 1.5 were considered to yield the final sample size of 474.

A multi-stage sampling technique was employed to select study participants. Three high schools were selected out of the seven found in the district using the lottery method. The number of sample points was determined by using the proportional allocation formula for each high school. School rosters were used as sampling frames, and 181, 241, and 40 participants were selected from Kolladiba, Chuahit, and Sankisa high schools, respectively by using the systematic sampling technique. Pregnant adolescents were excluded.

### Study variables and measurements

Dietary diversity practice was measured by minimum dietary diversity, and at least 5 varieties of food groups out of 10 in the past 24 h were considered as adequate intake. The composition includes (1) grains (white roots and tubers and plantains), (2) pulses (beans, peas, and lentils), (3) nuts and seeds, (4) dairy, (5) meat, (6) poultry and fish, (7) eggs, (8) dark green leafy vegetables, (9) other vitamin A-rich fruits and vegetables, and (10) other vegetables and fruits [17].

Anthropometric measurement (height and weight) was taken according to the World Health Organization (WHO) standard. Height was measured using a stadiometer and recorded to the nearest 0.1 cm. During the measurement, the prominent body parts of adolescent girls (occipital, shoulder, buttocks, and heel) touched the stadiometer; shoes were taken off, and girls were standing in the Frankfurt position. Weight was measured with the Seca beam scale balance and recorded to the nearest 0.1 kg. Heavy clothes and shoes were taken off. Body mass index (BMI) was measured using the AnthroPlus software. The wealth index was measured through the economic status of the household using principal component analysis with dimension reduction. Initially, coding was given for all variables between 0 and 1 based on their wealth status. Then, using principal component analysis (PCA), all the variables were entered into dimension reduction (factor analysis). The variables whose extraction value was less than 0.5 were removed step by step. All variables with extraction values greater than 0.5 were ranked into tertile of wealth status. Internal consistency or reliability of the tool was checked by Cronbach’s alpha. The distance to the nearest water source was measured in hours. In this case, a far distance was defined if the round trip distance from home to fetch water was more than 30 min. If the round trip took less than 30 min, it was categorized as near. Dietary diversity was measured using the FANTA 2016 1 day (24-h recall methods) diversity questionnaire. The diversity questionnaire used consisted of 10 food groups, which covered almost every food taken.

### Data collection tool, procedures, and quality control

A structured questionnaire, the anthropometric measurement for body mass index (BMI), and a standardized food security questionnaire from the Food and Nutrition Technical Assistance (FANTA) 2007 were used to collect data [18]. Socio-demographic factors (age, marital status, occupation, maternal educational level, religion, occupations, education status of father and mother, birth order, birth interval, ethnicity, and wealth index), selected health conditions (malaria, intestinal parasite, menorrhagia), and dietary diversity practice using 24-h recall methods in the school were assessed.

Mothers of adolescents were interviewed about socio-demographic and economic characteristics, household food security, wealth status, and environmental sanitation and hygiene practice through a face-to-face interview. The questionnaire was initially prepared in English and translated to Amharic and retranslated to English by language experts to check the consistency. The questionnaire was pretested on 5% of the study sample. Twelve Bachelor of Science (BSC) graduates (3 laboratory technicians and 9 clinical nurses) collected the data. Three health officers supervised the data collection. Two-day training was given to the data collectors and supervisors about the objectives, methodology of the study, and process of data collection by the principal investigator. Throughout the data collection, the collectors were supervised at each site, and regular meetings were held between data collectors, the supervisor, and the principal investigator. The daily collected data were checked for accuracy. Data cleaning and crosschecking were made by the principal investigator, and 10% of the data was double entered to check errors during entry.

### Data processing and analysis

All the filled questionnaires were checked manually for completeness and consistency. Data were coded and entered into EPI-info version 7 and exported to STATA version 14 for analysis. Descriptive statistics, including frequencies and proportions, were computed. Both bivariate and multivariate binary logistic regression analysis were performed. In the bivariate analysis, variables with a *p* value of < 0.2 were considered for the final model, and the adjusted odds ratio (AOR) with 95% confidence interval (CI) was used to show the presence and strength of associations. Finally, a *p* value of less than 0.05 in the multivariate logistic regression model was used to identify variables significantly associated with the dietary diversity practice. The goodness of fit test was carried out using the Hosmer and Lemeshow test.

### Ethical consideration

Ethical clearance was obtained from the Ethical Review Board of the University of Gondar. Written informed consent and assent was obtained from the selected adolescents and mothers or guardians. In the case of illiterate mothers, consent was documented by thumbprints on consent forms and signatures by a literate witness. All names and personal information were kept confidential, and the dataset was kept anonymous by using code numbers for analysis.

## Results

### The socio-demographic and economic status of respondents

The overall response rate in this study was 97.5%. The mean (± SD) ages of mothers and adolescents were 40.5 (39.8–41.2) and 16.6 (16.7–16.9) years, respectively. Among 462 participants, 430 (93.1%) were Orthodox Christians, and more than half (57.1%) were rural dwellers. The majority, 434 (93.9%), of mothers were married, and 272 (59.3%) unable to read and write. Of 458 respondents, 362 (79.0%) were housewives. Regarding the father’s educational level, 181 (40.8%) were not able to read and/or write, and more than half (60.1%) were farmers (Table 1).

**Table 1:** Sociodemographic characteristics of respondents in Dembia district, northwest Ethiopia, 2017 (*n* = 462)

Variable	Frequency	Percentage
**Religion**		
Orthodox	430	93.1
Muslim	32	6.9
**Residence**		
Urban	198	42.9
Rural	264	57.1
**Residency of adolescent**		
With-family	382	82.7
Away from family	80	17.3
**Living status of adolescent**		
With mother and father	369	79.9
With only mother	56	12.1
With only father	12	2.6
With guardian	18	3.9
With Others	7	1.5
**Marital status of the mother**		
Married	434	93.9
Divorced	17	3.7
Separated	4	0.9
Widowed	7	1.5
**Marital status of adolescent**		
Single	425	92.0
Married	34	7.4
Divorced	3	0.6
**Mother’s educational level**		
Cannot read and write	272	59.3
Can read and write	148	32.2
Primary	7	1.5
Secondary	7	1.5
Tertiary	25	5.5
**Mother’s occupation**		
Housewife	362	79.0
Civil servant	29	6.3
Farmer	21	4.6
Merchant	34	7.4
Labor	5	1.1
Private	3	0.7
No work	4	0.9
**Father’s occupation**		
Farmer	266	60.1
Craft	4	0.9
Merchant	51	11.5
Labor	14	3.2
Civil servant	40	9.0
NGO	11	2.4
Private	57	12.9
**Father’s education**		
Cannot read and write	181	40.8
Can read and write	233	50.2
Primary	3	0.7
Secondary	2	0.5
Tertiary	35	7.8
**Wealth status**		
Low	201	43.6
Middle	152	33.0
High	108	23.4

### Co-morbid condition of adolescents

Out of the total respondents, 29 (6.3%) of adolescents had chronic diseases. Of these, 16 (57.1%) had cardiac diseases, followed by 6 (21.4%) kidney and 6 (21.4%) tuberculosis. The remaining one adolescent had hypertension. Eighty-seven (18.8%) of adolescents had fever 2 weeks before the data collection. Of these, 52 (59.8%), 8 (9.2%), and 27 (31.0%) were due to malarial attack, typhoid, and unexplained diseases, respectively. Moreover, 23 (5%) adolescents had diarrhea, and of these, 17 (73.9%) visited a health center and received treatment. The main causes of diarrhea were hookworm which accounted for 26.1%, Ascaris for 39.1%, *Enterobius vermicularis* for 8.7%, and unspecified for 26.1% (Table 2).

**Table 2:** Co-morbid condition of adolescents in Dembia district, northwest Ethiopia, 2017 (*n* = 462)

Variable	Frequency	Percentage
**Known chronic diseases**		
No	433	93.7
Yes	29	6.3
**Fever (previous 2 weeks)**		
No	375	81.2
Yes	87	18.8
**Cause of fever**		
Not identified	27	31.0
Malaria	52	59.8
Typhoid	8	9.2
**Diarrheal episode**		
No	439	95.0
Yes	23	5.0
**Care at the health center for diarrhea**		
No	6	26.1
Yes	17	73.9
**Cause of diarrhea**		
Not identified	6	26.1
Hookworm	6	26.1
Ascaris	9	39.1
Enterobius	2	8.7
**Acute respiratory tract infection**		
No	306	66.2
Yes	156	33.8

### Dietary diversity practice and household food security of adolescents

Among the total respondents, 410 (88.74%) households were food secure. Injera, the main food item in the district, was utilized by 100% of the adolescents. Nearly two-thirds (66.2%) of the respondents ate Injera two times a day. Regarding fruit intake status, about 42.6% had no fruit intake and 20.0% had three times or more per day. Only 8 (1.7%) respondents consumed egg always, and 197 (42.6%) never ate eggs. Of the total respondents, 395 (85.5%) drank tea, and of these, nearly three-fourths (75.4%) drank after meals. Regarding soft drinks, 190 (41.1%) had at least one bottle per day. All adolescents consumed foods made from grains, cereals, roots, and tubers, while only 215 (46.6%) took dark green leafy vegetables. Also, 364 (78.8%) adolescents consumed no other vitamin A-rich and vegetable products (Table 3).

**Table 3:** Dietary practice of adolescents in Dembia district, northwest Ethiopia, 2017 (*n* = 462)

Variables	Frequency	Percentage
**Eating Injera per day(meal frequency)**		
Two times	85	18.4
Three times	306	66.2
Four times and more	71	15.4
**Frequency of eating citrus fruit per week**		
Once	99	21.4
Twice	70	15.2
Three times	11	2.4
Four times	85	18.4
None	197	42.6
**Frequency of eating egg**		
Always	8	1.7
Twice a week	43	9.3
Once a week	79	17.1
More than two times per month	32	6.9
One time per month	103	22.3
Never	97	42.7
**Frequency of using milk products**		
More than one time a day	19	4.1
One time a day	102	22.8
One time per week	130	28.1
Never	211	45.7
**Drinking of tea or coffee**		
No	67	14.5
Yes	395	85.5
**Timing of drinking of tea or coffee**		
Before	97	24.6
After	298	75.4
**Drinking of soft drinks per week**		
No	272	58.9
Yes	190	41.1
**How many soft drinks drink**		
Once	104	54.7
Twice	35	18.4
Three times	37	19.5
Four and above	14	7.4
**Dark green leafy vegetables**		
No	246	53.4
Yes	215	46.6
**Another vitamin A-rich and vegetable**^**a**^		
No	364	78.8
Yes	98	21.2
**Other fruit**		
No	338	73.2
Yes	124	26.8
**Organ meat**		
No	456	98.7
Yes	6	1.3
**Meat and fish**		
No	443	95.9
Yes	19	4.1

^a^ Other vitamin A-rich and vegetables: carrots, sweet potatoes, green leafy veggies, pumpkin, liver, parsley and other herbs, milk, fish, tomato, and red bell pepper

### Environmental characteristics of respondents

Two hundred seventy-nine (60.4%) and 120 (26.0%) of the adolescents used communal and pipe water, respectively. More than two-thirds, 67.5%, of the respondents did not use any type of water treatment. Nearly one-third (29.7%) and 13 (2.8%) used water agar and boiling, respectively for treating water. Of the total, 375 (81.2%) had a toilet. Almost all (99.1%) practiced handwashing either before or after eating and before or after food processing (Table 4).

**Table 4:** Environmental characteristics of respondents in Dembia district, northwest Ethiopia, 2017 (*n* = 462)

Variables	Frequencies	Percentages
**Source of water**		
Protected	19	4.1
River	9	2.0
Unprotected	16	3.4
Pond	19	4.1
Pipe	120	26.0
Communal	279	60.4
**Treatment of water**		
Boiling	13	2.8
Water agar	137	29.7
Never	312	67.5
**Availability of toilet**		
No	87	18.8
Yes	375	81.2
**The practice of handwashing**		
No	4	0.9
Yes	458	99.1
**Handwashing before a meal**		
No	26	5.6
Yes	436	94.4
**Handwashing after the meal**		
No	28	6.1
Yes	434	93.9
**Handwashing after toilet**		
No	209	45.2
Yes	253	54.8
**Handwashing before food processing**		
No	210	45.5
Yes	252	54.5
**Handwashing after food processing**		
No	55	11.9
Yes	407	88.1
**The distance of water (h)**		
Near	459	99.4
Far	3	0.6

### Prevalence of dietary diversity practice

Of the total respondents, 149 (32.3%) (95% CI 27.9, 36.8) reported adequate dietary practices.

### Factors associated with dietary diversity among adolescents

In the bivariate logistic regression analysis, religion, residence, adolescent age, father’s occupation, birth order, frequency of meals, source of water, treatment of water, availability of toilet, BMI, and wealth status were factors associated with dietary diversity at a *p* value of less than 0.2. Consequently, these variables were included in multivariate logistic regression analysis, and it was noted that religion, father’s occupation, BMI, and wealth status were significantly associated with dietary diversity at a *p* value of < 0.05.

The odds of inadequate dietary diversity among Muslims were 70% lower compared with that of Orthodox Christians (AOR = 0.3; 95% CI 0.1–0.7). Similarly, the odds of inadequate dietary diversity among self-employed decreased by 70% compared to farmers (AOR = 0.3; 95% CI 0.1–0.9). The lower odds of inadequate dietary diversity were observed among middle (AOR = 0.5; 95% CI 0.3–0.8) and richest (AOR = 0.3; 95% CI 0.2–0.6) wealth status compared with poor families. Furthermore, the odds of inadequate dietary diversity among underweight adolescents were 3.5 times higher compared to those of well-nourished (AOR = 3.5; 95% CI 1.3–9.5) (Table 5).

**Table 5:** Factors associated with dietary diversity practice among adolescents in Dembia district, northwest Ethiopia, 2017 (*n* = 462)

Variables	Frequency		COR 95% CI	AOR 95% CI
	Yes	No		
**Religion**				
Orthodox	126	304	1	1
Muslim	23	9	0.2 (0.1, 0.4)	0.3 (0.1–0.7)
**Residency**				
Urban	88	110	1	1
Rural	61	203	2.7 (1.8, 4.0)	0.9 (0.4–2.5)
**Adolescent age**				
15–16	75	125	1	1
17–19	74	188	1.5 (1.0, 2.7)	1.5 (0.9–2.4)
**Father’s occupation**				
Farmer	63	218	1	1
Merchant	24	27	0.3 (0.2, 0.6)	0.7 (0.2–1.9)
Civil servant	21	19	0.3 (0.1, 0.5)	0.4 (0.1–1.1)
Private	38	44	0.3 (0.2, 0.6)	0.3 (0.1–0.9)
**Birth order**				
1–4	117	212	1	1
5–10	32	101	1.7 (1.1, 2.8)	1.4 (0.8–2.3)
**Wealth index**				
Low	41	160	1	1
Middle	60	92	0.4 (0.2–0.6)	0.5 (0.3–0.8)
High	48	60	0.3 (0.2–0.5)	0.3 (0.2–0.6)
**BMI for age**				
Normal	143	278	1	1
Underweight	6	35	0.5 (0.2–0.9)	3.5 (1.3–9.5)
**Availability of latrine**				
No	36	51	1	1
Yes	215	160	1.9 (1.1–3.3)	1.8 (0.8–39.7)
**Frequency of meal**				
2	24	61	1	1
3	98	208	0.8 (0.5–1.4)	1.1 (0.6–1.9)
4	27	44	0.6 (0.3–1.3)	0.8 (0.4–1.6)
**Treatment of water**				
Boiling	7	6	1	1
Water agar	90	47	1.6 (0.5–5.1)	1.0 (0.3–4.0)
Never	245	67	3.1 (1.0–9.5)	1.8 (0.5–6.7)

## Discussion

Even though adolescents are considered as typically low-risk groups for poor health, many health problems later in life can be improved or avoided by adopting healthy lifestyle habits among adolescence [19]. Ethiopian school children and adolescents constitute > 35% of the total population among the most affected groups [20].

The overall prevalence of adequate dietary diversity in this study was 32.3% (95% CI; 27.9, 36.8). The finding is in line with a study conducted in Zimbabwe in which 33% of adolescents reported that they consumed a diverse diet [21]. However, the estimate in this study is lower compared to the study conducted in India where more than 50% of adolescents had access to dietary diversity. The possible reason could be a wider distribution of food variety in India. Similarly, our estimate is lower when compared to studies conducted elsewhere in Ethiopia, for example, studies reported 74.7% and 38.7% in Jimma town in 2016 and 2017, respectively, and 41.2% in Adama, 80.7% in Bale zone, and 45.3% in Gobe town. Possible explanations for these variations could be the economic difference and the fact that only starchy foods are commonly consumed in those settings.

On the other hand, our estimate is higher than a study conducted in the Gurage zone [16] and the Amhara region, Ethiopia [22], which reported a prevalence of 26.8% and 21.8%, respectively. The possible reason could be in defining the outcome variable, and the observation that nearly half of the households were food insecure. In the former study, the outcome was measured using a tool having nine questions, and dietary diversity was defined as eating 4 and above varieties of food.

The odds of having inadequate dietary diversity were 70% lower among Muslims compared to Orthodox Christians. The possible explanation could be the difference in residence. This is because in this study all Muslim participants lived in urban settlements while almost the majority of the Christians were found in rural areas. According to a study conducted in the Gurage zone, the urban residence was associated with good dietary diversity practices, perhaps because urban settings provide better access to the selection of foods [16].

The odds of facing inadequate dietary diversity were 70% lower among adolescents with self-employed fathers compared to farmers. This could be due to a better economic status of those self-employed. The majority of participants in our study were urban dwellers, and this might create more access to different types of food. Inversely, this finding is in contrast to that of a study conducted in Jimma, where farmers were 56% more likely to consume diversified diets compared to other workers [23].

The odds of encountering inadequate dietary diversity decreased by 70% and 50% among the richest and middle wealth quartiles compared to the poor wealth quartile, respectively. This finding is supported by studies conducted in Iran and Ethiopia (Gurage zone and Jimma town) where a significant association was noted between dietary diversity score (DDS) and the economic status of the families [18, 24]. This could be because often poor people do not have adequate access to diversified foods.

The odds of having inadequate dietary diversity among underweight adolescents were 3.5 times higher compared to the well-nourished. The finding was supported by that of a study conducted in Karnataka, India, where it was found that there was a relationship between DDS and BMI which evidenced that subjects with a DDS of 5 and above had better odds of having their BMI in the normal range [25], but if there was lack of diversified foods, the individual had a higher chance of becoming malnourished. This finding was supported by a study in Iran [26, 27] which showed that those adolescent girls who had a higher BMI associated with adequate dietary diversity. However, the study conducted in the Gurage zone revealed that higher DDS was not significantly associated with normal BMI [16]. The study will be generalized to all female adolescents in Dembia district. This study shares the typical limitations of a cross-sectional study. As a result, it does not show as temporal relationship between the outcome and independent variables. Moreover, this study may be affected by recall bias in reporting of eating behavior.

## Conclusion

The findings of this study showed that one-third of the adolescent girls had adequate dietary diversity; father’s occupation, religion, wealth status, and nutritional status of the adolescents were significantly associated with dietary diversity. Low level of dietary diversification suggested the need for strengthening efforts to improve the dietary practice of adolescents by giving due attention to poor households and undernourished adolescents.

## Data Availability

Data will be available upon request from the corresponding author.

## Abbreviations

AOR: Adjusted odds ratio
ANC: Antenatal care
BMI: Body mass index
CI: Confidence interval
COR: Crude odds ratio
EDHS: Ethiopian Demographic Health Survey
EPI: Expanded program on immunization
FANTA: Food and Nutrition Technical Assistance
MDG: Millennium Development Goal
NGO: Nongovernmental organization
SAM: Severe acute malnutrition
SUN: Scale up nutrition
UNICEF: United Nations Children Emergency Fund
WHO: World Health Organization

## References

[1] Yi Y, Turney K, Wildeman C. Mental health among jail and prison inmates. Am J Men's Health. 2017;11(4):900–9. 10.1177/1557988316681339 PMC5675352 27932588

[2] Bowler N, Phillips C, Rees P (2018). The association between imported factors and prisoners’ mental health: implications for adaptation and intervention. Int J Law Psychiatry.

[3] Gonçalves LC, Endrass J, Rossegger A, Dirkzwager AJ (2016). A longitudinal study of mental health symptoms in young prisoners: exploring the influence of personal factors and the correctional climate. BMC Psychiatry.

[4] Forrester A, Hopkin G (2019). Mental health in the criminal justice system: a pathways approach to service and research design. Crim Behav Ment Health.

[5] Helms R, Gutierrez RS, Reeves-Gutierrez D (2016). Jail mental health resourcing: a conceptual and empirical study of social determinants. Int J Offender Ther Comp Criminol.

[6] Bundy DA, Shaeffer S, Jukes M, Beegle K, Gillespie A, Drake L (2006). School based health and nutrition programs. Dis Control Priorities Dev Ctries.

[7] Peters LW, Kok G, Ten Dam GT, Buijs GJ, Paulussen TG (2009). Effective elements of school health promotion across behavioral domains: a systematic review of reviews. BMC Public Health.

[8] Forrester A, Till A, Simpson A, Shaw J (2018). Mental illness and the provision of mental health services in prisons. Br Med Bull.

[9] Alemayehu F, Ambaw F, Gutema H (2019). Depression and associated factors among prisoners in Bahir Dar Prison, Ethiopia. BMC Psychiatry.

[10] Martin MS, Crocker AG, Potter BK, Wells GA, Grace RM, Colman I (2018). Mental health screening and differences in access to care among prisoners. Can J Psychiatry.

[11] Yoon IA, Slade K, Fazel S (2017). Outcomes of psychological therapies for prisoners with mental health problems: a systematic review and meta-analysis. J Consult Clin Psychol.

[12] O'Toole S, Maguire J, Murphy P (2018). The efficacy of exercise referral as an intervention for Irish male prisoners presenting with mental health symptoms. Int J Prison Health.

[13] Goomany A, Dickinson T (2015). The influence of prison climate on the mental health of adult prisoners: a literature review. J Psychiatr Ment Health Nurs.

[14] Baidawi S (2016). Older prisoners: psychological distress and associations with mental health history, cognitive functioning, socio-demographic, and criminal justice factors. Int Psychogeriatr.

[15] Macdonald M (2013). Women prisoners, mental health, violence and abuse. Int J Law Psychiatry.

[16] Worku M, Hailemicael G, Wondmu A (2017). Dietary diversity score and associated factors among high school adolescent girls in Gurage Zone, Southwest Ethiopia. World J Nutr Health.

[17] The Food and Agriculture Organization of the United Nations and USAID’s Food and Nutrition Technical Assistance III Project (FANTA) mbF. Minimum dietary diversity for women a guide to measurement. Rome: University of California; 2016.

[18] Adraro W, Kerebih H, Tesema W, Abamecha F, Hailesilassie H (2019). Nearly three in every five prisoners experience common mental disorders (CMDs) in Jimma correctional institution; south-West Ethiopia. BMC Public Health.

[19] Wali A. Nutrition for your adolescent. Wali’s Nutrition. Available from: https://waliangela.wordpress.com/2015/05/06/nutrition-for-your-adolescent/. Cited 2015 Dec 11.

[20] Population Reference Bureau (2013). The world’s youth 2013 data sheet, 1–17. Retrieved from http://www.prb.org/Publications/Datasheets/2013/youth-datasheet-2013.aspx/.

[21] Masterson AR (2015). Assessment of adolescent girl nutrition, dietary practices, and roles in Zimbabwe Mangwe and Tsholotsho Districts.

[22] Wassie MM, Gete AA, Yesuf ME, Alene GD, Belay A, Moges T (2015). Predictors of nutritional status of Ethiopian adolescent girls: a community based cross sectional study. BMC Nutr.

[23] Tamiru D, Argaw A, Gerbaba M, Nigussie A, Ayana G, Belachew T (2016). Improving dietary diversity of school adolescents through school based nutrition education and home gardening in Jimma Zone: quasi-experimental design. Eat Behav.

[24] Melaku Y, Dirar A, Feyissa GT, Tamiru D. Optimal dietary practices and nutritional knowledge of school adolescent girls in Jimma Town, South West Ethiopia. Int J Adolesc Youth. 2018;23(3):299–307.

[25] Shashikantha S, Sheethal M, Vishma B (2017). Dietary diversity among women in the reproductive age group in a rural field practice area of a medical college in Mandya district, Karnataka, India. Int J Community Med Public Health.

[26] Vakili M, Abedi P, Sharifi M, Hosseini M (2013). Dietary diversity and its related factors among adolescents: a survey in Ahvaz-Iran. Glob J Health Science.

[27] Solomon A, Mihretie G, Tesfaw G (2019). The prevalence and correlates of common mental disorders among prisoners in Addis Ababa: an institution based cross-sectional study. BMC Res Notes.

